# Morphological MR imaging of the articular cartilage of the knee at 3 T—comparison of standard and novel 3D sequences

**DOI:** 10.1007/s13244-015-0405-1

**Published:** 2015-04-09

**Authors:** Pieter Van Dyck, Floris Vanhevel, Filip M. Vanhoenacker, Kristien Wouters, David M. Grodzki, Jan L. Gielen, Paul M. Parizel

**Affiliations:** 1 Department of Radiology, Antwerp University Hospital and University of Antwerp, Wilrijkstraat 10, 2650 Antwerp (Edegem), Belgium; 2 Department of Radiology, Ghent University Hospital and University of Ghent, De Pintelaan 185, 9000 Ghent, Belgium; 3 Department of Radiology, AZ St-Maarten, Rooienberg 25, 2570 Antwerp (Duffel), Belgium; 4 Department of Biostatistics, Antwerp University Hospital and University of Antwerp, Wilrijkstraat 10, 2650 Antwerp (Edegem), Belgium; 5 Siemens Healthcare, Allee am Röthelheimpark 2, 91052 Erlangen, Germany

**Keywords:** Knee, Magnetic resonance imaging, Cartilage, 3 T-isotropic

## Abstract

**Objectives:**

This study sought to compare various 3D cartilage sequences and to evaluate the usefulness of ultrashort TE (UTE) imaging, a new technique to isolate signal from the osteochondral junction.

**Methods:**

Twenty knees were examined at 3 T with 3D spoiled GRE (FLASH), double-echo steady-state (DESS), balanced SSFP, 3D turbo spin-echo (TSE), and a prototype UTE sequence. Two radiologists independently evaluated all images. Consensus readings of all sequences were the reference standard. Statistical analysis included Friedman and pairwise Wilcoxon tests. Retrospective analysis of UTE morphology of osteochondral tissue in normal and abnormal cartilage seen at conventional MR was also performed.

**Results:**

Three-dimensional TSE was superior to other sequences for detecting cartilage lesions. FLASH and DESS performed best in the subjective quality analysis. On UTE images, normal cartilage exhibited a high-intensity linear signal near the osteochondral junction. Retrospective analysis revealed abnormal UTE morphology of the osteochondral junction in 50 % of cartilage lesions diagnosed at conventional MR.

**Conclusions:**

Cartilage imaging of the knee at 3 T can be reliably performed using 3D TSE, showing high accuracy when compared to standard sequences. Although UTE depicts signal from the deep cartilage layer, further studies are needed to establish its role for assessment of cartilage.

***Main Messages*:**

• *MRI is the best available imaging method for assessment of knee cartilage*.

• *Cartilage imaging can be reliably performed using 3D TSE*.

• *UTE cannot be used as a single sequence to assess cartilage*.

## Introduction

Magnetic resonance (MR) imaging is generally regarded as the best available noninvasive method for evaluating injury and repair of the articular cartilage of the knee [1–3]. Currently, most evaluation of knee cartilage is done with two-dimensional (2D) acquisition techniques, such as turbo spin-echo (TSE) sequences, as they provide excellent tissue contrast and high in-plane spatial resolution [4]. However, 2D sequences have relatively thick slices (≥2 mm) and small gaps (0.2 mm) between slices, which can obscure pathology because of partial volume averaging [4, 5].

Three-dimensional (3D) imaging has the great potential of acquiring volumetric data sets with isotropic resolution, providing thin (0.5–0.6 mm) contiguous slices through the knee joint, thereby reducing partial volume artefacts. Multiplanar reformats along any user-defined imaging plane can be generated from the source data without loss of resolution [4–6].

Techniques for morphological 3D MR imaging of cartilage have changed rapidly in the past 2 decades. Gradient-echo (GRE) sequences were the first 3D sequences used for cartilage imaging [4, 7, 8]. They are classically divided into dark fluid sequences (e.g. spoiled gradient echo or FLASH) and bright fluid sequences (e.g. double echo steady-state or DESS) based upon the signal intensity of synovial fluid [4, 8]. These traditional GRE methods have been shown to be highly accurate for cartilage lesions in various studies and are still considered the standard of reference for high-resolution 3D isotropic imaging of cartilage [5–8]. A water-excited DESS sequence has been used in various knee osteoarthritis trials and is currently being used in the Osteoarthritis Initiative to assess the articular cartilage of the knee joint [9]. Many different methods of steady-state free-precession-based imaging (SSFP), a new variant of the GRE method, are also available for imaging cartilage, and all have higher cartilage signal compared with conventional GRE methods [10]. However, major disadvantages of 3D GRE imaging include their sensitivity to susceptibility artefacts and their suboptimal tissue contrast [4–8].

The increased availability of high-field 3-T imaging systems combined with improved coil technology facilitate the use of novel isotropic 3D sequences in clinical practice. Recently, 3D TSE sequences (e.g. SPACE) were developed for 3-T scanners. The important advantage of the 3D TSE acquisitions is their capability of mimicking the contrast properties of conventional 2D TSE intermediate-weighted acquisitions, allowing for better tissue contrast and higher conspicuity of cartilage lesions [5, 11].

In the knee, the superficial and middle layers of articular cartilage have T2 values of ~50 ms, while the deep cartilage layer has much shorter T2 values, in the range of 1 to 2 ms or less [12]. Therefore, with a practical minimum echo time (TE) of about 10 ms for a spin-echo (SE) sequence and of about 2 ms for a GRE sequence, conventional MR imaging lacks the ability to display signal near the osteochondral junction. Through the use of ultrashort TE (UTE) sequences, now becoming available on clinical 3-T scanners, the minimum TE can be considerably reduced and the signal detected before it has totally decayed. As a result, signal can now be acquired from these previously ‘invisible’ tissues at the bone-cartilage interface, allowing for direct visual assessment [12, 13].

Controversy remains as to which is the single best 3D sequence for clinical cartilage imaging of the knee at 3 T [14, 15]. Also, clinical UTE imaging of the knee has been performed in healthy volunteers [16, 17], but studies investigating the role of morphological UTE imaging in patients with cartilage lesions are lacking. Thus, the main purpose of the present study was to compare standard and novel 3D cartilage MR sequences at 3 T. It was hypothesised that 3D TSE acquisitions compare favourably to the standard of reference GRE sequences for clinical cartilage imaging. In addition, we sought to explore how the normal UTE signal patterns change in normal and abnormal cartilage seen at conventional MR.

## Materials and methods

### Patient selection

This prospective study was approved by the Institutional Review Board of the Antwerp University Hospital according to the ICH Good Clinical Practice rules (registration no. B300201421002). Written informed consent was obtained from all of the participants after the nature of the examinations had been fully explained. From 1 March to 31 May 2014, a total of 20 subjects were prospectively enrolled in this study, including 10 healthy volunteers (8 males and 2 females; mean age 25 years; range 23–32 years; mean body mass index 22.6 kg/m^2^) and 10 patients (2 males and 8 females; mean age 49 years; range 27–61 years; mean body mass index 26.4 kg/m^2^). All volunteers were medical or paramedical personnel (e.g. medical students, residents, and nurses), and none of them had known musculoskeletal disease or prior knee symptoms. A patient was enrolled in the study if they (1) had a clinical suspicion of a cartilage abnormality at the knee (pain and stiffness for more than 6 months, n = 5; anterior knee pain aggravating when climbing stairs, n = 3; recent episodes of pain and functional limitation in nonprofessional marathon runners, n = 2), (2) had no history of prior knee surgery, and (3) consented to undergo a 3-T MRI of the knee with dedicated cartilage imaging sequences. Subjects with contra-indications to the use of MRI (e.g. aneurysm clip, cardiac pacemaker, claustrophobia) were not included in the study. The right knee was imaged in all study participants.

### MR imaging protocol

**Fig. 1:**
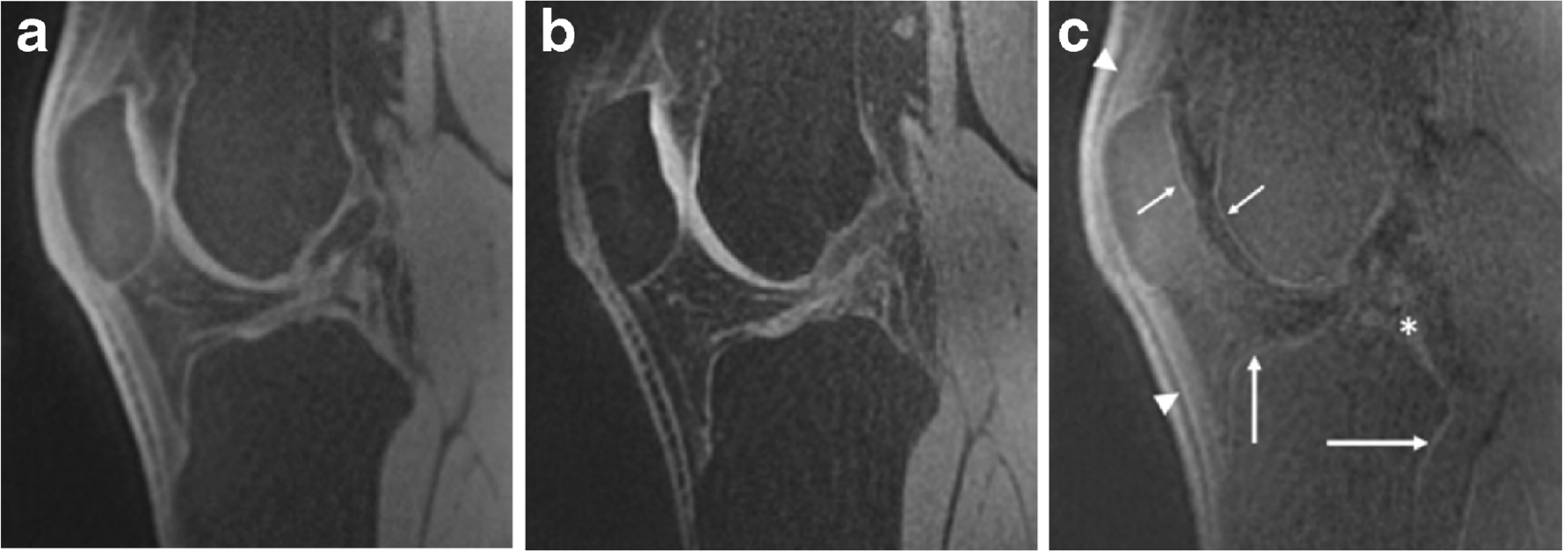
UTE imaging of the articular cartilage of the knee in a 24-year-old healthy volunteer. Two echos with varying TE were acquired. **a** First echo, obtained at a minimum TE (=60 μs), and **b** second echo, obtained at longer TE (=3.92 ms). The second echo image was then digitally subtracted (**c**) from the first one to suppress signal from the long T2 tissue components, improving visualisation of the deep layer of cartilage. UTE subtraction image shows distinct high-intensity linear signal near the osteochondral junction at the patella and trochlea (small arrows). Also note bright signal at the cortical margins (large arrows), extensor apparatus (arrowheads), and posterior cruciate ligament (*)

All images were acquired with a 3-T MR imaging system (Magnetom SKYRA, Siemens, Erlangen, Germany) by using a transmit/receive 15-channel knee coil (Quality Electrodynamics, Mayfield, OH, USA). The MR protocol consisted of five different isotropic 3D sequences: water-excited (we) 3D Fast-Low Angle Shot (FLASH), we Double-Echo Steady-State (DESS), we True Fast Imaging with Steady-State Free Precession (TrueFISP), fat-saturated (fs) intermediate-weighted 3D Turbo Spin-Echo Sampling Perfection with Application optimised Contrasts using variable flip angle Evolutions (SPACE), and a prototype 3D Ultrashort Echo-Time (UTE) sequence. All sequences had a sagittal plane of acquisition and used a 0.6 mm^3^ voxel volume, except for the 3D UTE sequence, which employed a 0.8 mm^3^ voxel size. Detailed parameters for all imaging sequences are provided in Table 1. In our study, a dual echo 3D UTE sequence was used consisting of a 60-μs nonselective RF pulse followed by 3D radial ramp sampling from the centre to the surface of a sphere. In order to achieve the shortest possible TE, data acquisition started already during ramp-up time of the readout gradient. Frequency-based fat suppression was available as an additional option. Difference images were formed to suppress signals from long T2 components by subtracting the later echo time (TE = 3.92 ms) images from the first image (TE = 60 μs) (Fig. 1). The scan time of the 3D UTE sequence was 6 min 48 s, which is feasible for routine clinical use.

**Table 1:** MRI parameters for 3D sequences

	3D cartilage imaging sequence				
Imaging parameter^*^	FLASH	DESS	TruFISP	SPACE	UTE
TR (ms)	10.1	14.8	7.18	1200	10
TE (ms)	4.92	5.0	3.18	30	0.06/3.92
Acquistion matrix	256 × 233	256 × 240	256 × 240	256 × 256	256 × 256
FOV (mm)	150 × 150	150 × 140	150 × 140	160 × 160	200 × 200
Flip angle	10	25	28	120	15
Averages	1	1	3	1	1
PAT	2	2	2	2	no
No. of slices	160	160	192	192	256
Imaging time (min:s)	3:02	3:42	9:09	10:04	6:48

^*^TR = repitition time; TE = echo time; FOV = field of view; PAT = parallel acquisition technique

### MR imaging analysis

All images were independently reviewed during separate sessions by two readers [with 25 years (reader A) and 10 years (reader B) of experience in musculoskeletal radiology at the time of the study]. Images were obtained without any annotation. At the time of review, the readers were blinded to the patient’s clinical history and clinical findings. A total of 100 MRI data sets (5 data sets per subject for a total of 20 study subjects) were reviewed in 5 separate sessions at least 96 h apart to prevent recall bias. Each session consisted of 20 data sets that were presented randomly except that different data sets of the same patient were never reviewed in the same session. The readers used sagittal source data to create multiplanar reformatted (MPR) images in the axial and coronal plane. Subjective image quality was assessed by using the following criteria: edge sharpness, artefacts, amount of noise, tissue contrast, and reformat quality. The quality score consisted of an ordinal 5-point Likert-scale and was defined as (1) poor, unacceptable for diagnostic purposes; (2) adequate but poorer than average quality; (3) average quality of a diagnostic acceptable image; (4) above average quality; (5) best quality. The readers graded each of the six articular surfaces of the knee as follows: (1) normal; (2) low-grade cartilage defect (<50 % of total cartilage thickness); (3) high-grade cartilage defect (>50 % of total cartilage thickness). Each reader was asked to assign a confidence level to the diagnosis as follows: (1) definitely normal; (2) probably normal; (3) probably abnormal; (4) definitely abnormal. Presence of bone marrow oedema was also recorded using the same 4-point Likert scale. On UTE images, the readers prospectively evaluated the morphology of the deep osteochondral tissues to predict the status of the overlying cartilage: a distinct, continuous high linear signal at the cartilage/bone interface was considered normal (indicating normal cartilage) and thinning, interruption, or complete absence of that signal was considered abnormal (indicating cartilage lesion), according to previously described criteria by Bae et al [12, 18]. Finally, the UTE morphology of the osteochondral tissue was retrospectively assessed in consensus and with knowledge of the findings on conventional MR sequences.

### Standard of reference

The standard of reference for the MR abnormalities was based on image analysis by two senior radiologists in consensus with all available MR sequences as well as clinical findings. Both radiologists had more than 25 years of experience in musculoskeletal radiology at the time of the study and were not involved in the blinded study readings.

### Statistical analysis

Statistical analysis was performed by a statistician with use of R software (version 3.1.1.; R Foundation for Statistical Computing, Vienna, Austria). Differences between the MR sequences were considered to be statistically significant if *p* < 0.05. The Friedman test and pairwise Wilcoxon tests with Bonferroni-Holm correction for multiple testing were used to compare the performance and subjective image quality gradings of all sequences [19]. Interreader agreement was determined using the weighted kappa (к) statistic with к value ≥ 0.8 indicating excellent agreement. To increase statistical power for the performance analysis, calculations were made for all articular surfaces, all grades of severity, and both readers combined. Therefore, a bootstrapping procedure with 1000 samples at the level of the patients was used to calculate standard errors. Finally, the proportion of definite MR diagnoses (i.e. definitely normal or definitely abnormal) was calculated for each sequence.

## Results

### Diagnostic performance and confidence analysis

**Fig. 2:**
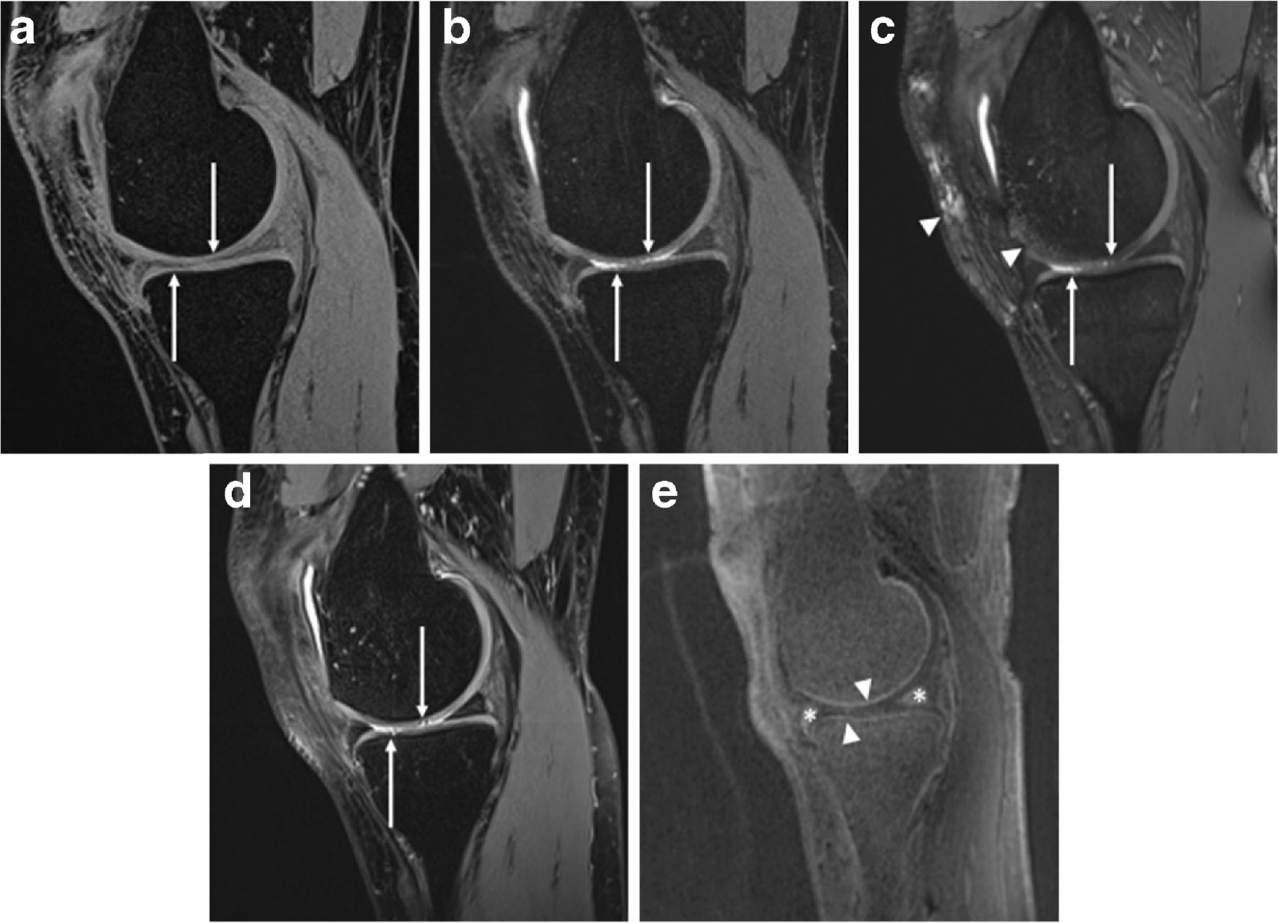
A 32-year-old female marathon runner with episodes of medial knee pain. Comparison of sagittal FLASH (**a**), DESS (**b**), TruFISP (**c**), and SPACE (**d**) showing cartilage defects (arrows) at the medial knee compartment. Lesion conspicuity is highest on SPACE sequence. Susceptibility artefacts are seen on TruFISP (arrowheads). UTE subtraction image (**e**) shows normal appearance of deep osteochondral tissue at the medial knee compartment (arrowheads). Also note that, whereas the meniscus appears dark on conventional sequences, bright meniscal signal is seen on the UTE image (*)

**Fig. 3:**
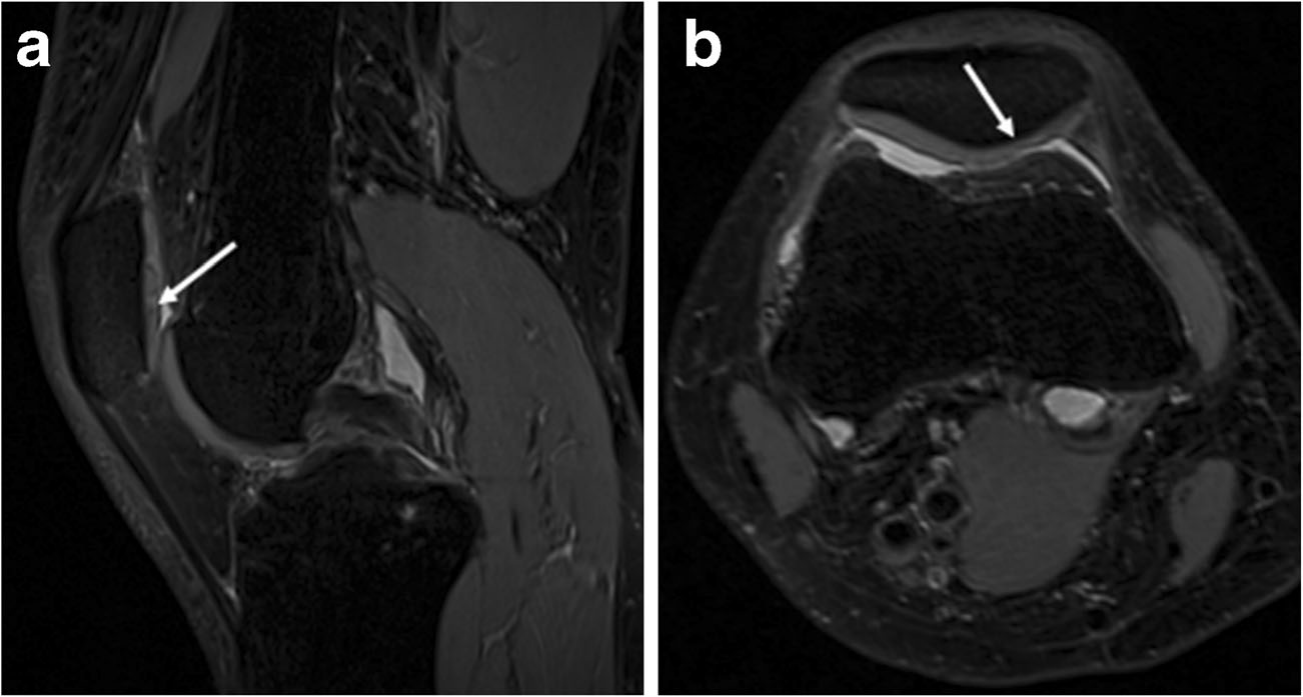
A 46-year-old female patient with episodes of anterior knee pain. **a** Sagittal SPACE image shows signal heterogeneity (arrow) at the patellar cartilage. **b** Axial reformatted SPACE image shows superficial cartilage loss on the medial patellar facet (arrow)

Consensus readings of all available MR sequences revealed 20 cartilage defects (low grade <50 %, n = 7; high grade >50 %, n = 13) in 10 patients (medial tibia, n = 2; lateral tibia, n = 1; medial femoral condyle, n = 5; lateral femoral condyle, n = 1; patella, n = 9; trochlea, n = 2). Since 120 articular cartilage surfaces were each evaluated by 2 readers, we had a total of 240 individual surface interpretations. A summary of the performance of all sequences in detecting cartilage defects is given in Table 2. For the detection of cartilage lesions, the highest sensitivity was achieved with SPACE (98 %), being significantly higher than TruFISP (sensitivity = 55 %, p = 0.014) and UTE (sensitivity = 20 %, p = 0.010) (Figs. 2 and 3). There were no significant differences in specificity and accuracy for diagnosing cartilaginous lesions among FLASH, DESS, TruFISP, and SPACE (all values ≥90 %). Accuracy of UTE was 86 %, being significantly lower than FLASH (accuracy = 95 %, p = 0.010) and DESS (accuracy = 94 %, p = 0.036).

**Table 2:** Performance of 3D sequences in detecting cartilage defects

Sequence	Sensitivity	SE^*^	Specificity	SE*	Accuracy	SE*
FLASH	0.80 (32/40)	0.09	0.98 (195/200)	0.01	0.95 (227/240)	0.02
DESS	0.68 (27/40)	0.13	0.99 (198/200)	0.01	0.94 (225/240)	0.03
TruFISP	0.55 (22/40)	0.13	0.97 (194/200)	0.02	0.90 (216/240)	0.03
SPACE	0.98 (39/40)	0.03	0.96 (192/200)	0.02	0.96 (231/240)	0.01
UTE	0.20 (8/40)	0.08	0.99 (198/200)	0.01	0.86 (206/240)	0.04

*SE = standard error

Note: Data are given for all articular surfaces, all grades of cartilage lesion severity, and both readers combined

A review of the radiologists’ confidence scores in diagnosing cartilage lesions revealed that readers were most confident with DESS sequence (88 % definite diagnoses for both readers). For the detection of subchondral bone marrow oedema, the readers were most confident with SPACE (95 % definite diagnoses for both readers).

### Image quality analysis

There were no significant differences in overall image quality among FLASH, DESS, and SPACE, but all three performed significantly better than TruFISP and UTE (p < 0.0001) (Table 3). Mild blurring artefacts were seen with the SPACE sequence; TruFISP image quality was degraded by susceptibility artefacts. Although UTE images exhibited high signal intensity at the osteochondral junction in all subjects, the UTE sequence was ranked lowest for contrast at the cartilage/bone interface because of poor image quality and blurring artefacts. DESS, TruFISP, and SPACE performed significantly better than FLASH (p ≤ 0.0001) for separating between the surface layer of the articular cartilage and joint fluid. There were no significant differences in reformat quality among FLASH, DESS, and SPACE. Agreement between readers for the subjective assessment of overall image quality was excellent (к = 0.90).

**Table 3:**
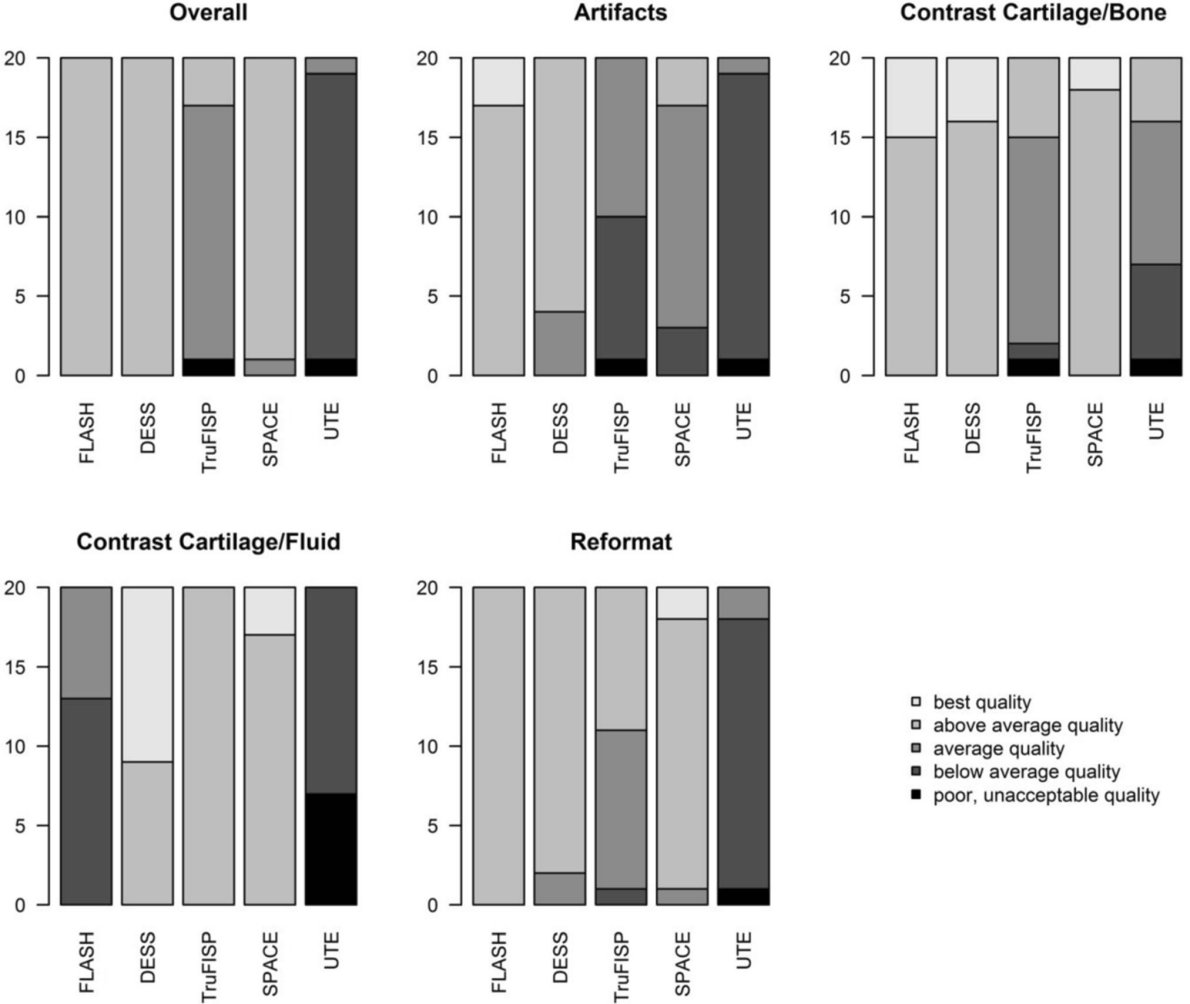
Qualitative image analysis

### Analysis of UTE morphology of the osteochondral junction

**Fig. 4:**
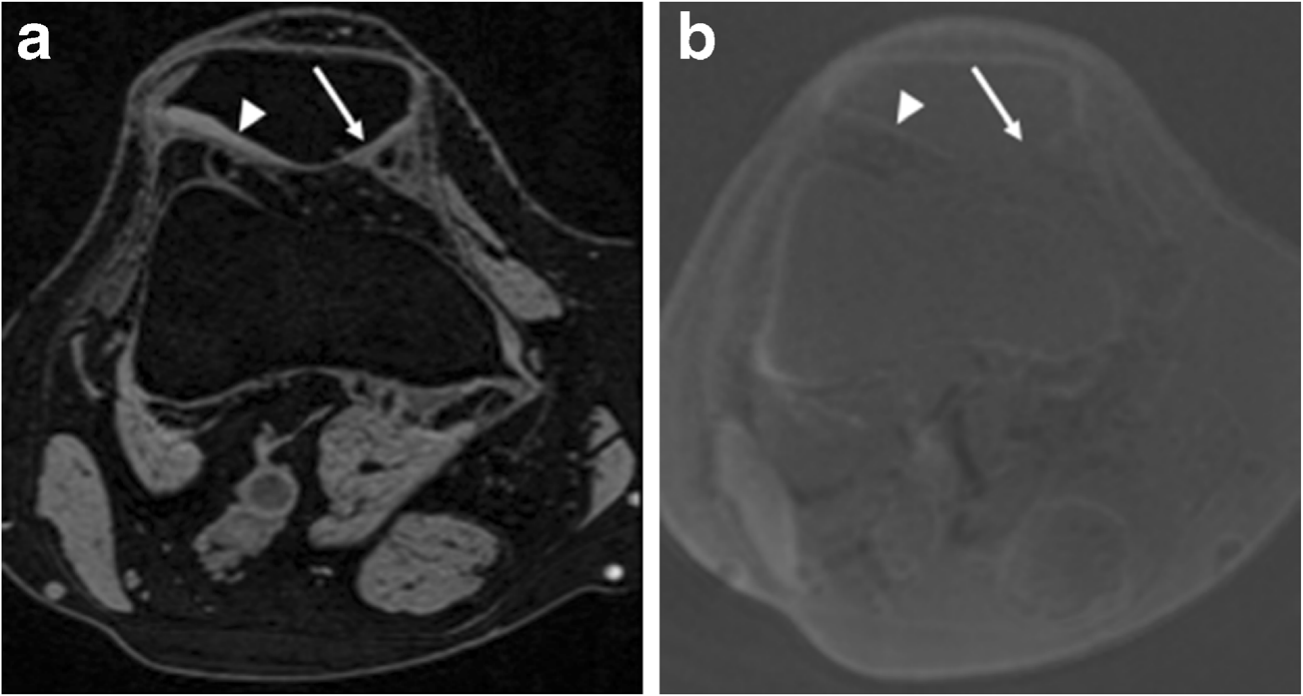
A 59-year-old female with chronic knee pain. **a** Axial reformatted FLASH image shows extensive cartilage loss at the medial patella (arrow). Normal cartilage is seen on the lateral patellar facet (arrowhead). **b** Axial reformatted UTE subtraction image shows absence of high-intensity linear signal near the osteochondral junction at the medial patella (arrow). Normal UTE appearance of the osteochondral junction on the lateral patellar facet is demonstrated (arrowhead)

On UTE images, normal cartilage exhibited a high-intensity linear signal near the osteochondral junction. Retrospective analysis of UTE morphology of the osteochondral junction in subjects diagnosed with cartilage lesions (n = 20) on conventional MRI revealed ten abnormalities (irregular, n = 5; disruption, n = 4; absence, n = 1). Of these, six were diagnosed with high-grade cartilage lesions and four with low-grade cartilage lesions on conventional MRI (Fig. 4). Ten cartilage lesions showed a normal appearance of the osteochondral junction on UTE images. Of these, seven were diagnosed with high-grade cartilage lesions and three with low-grade cartilage lesions on conventional MRI (Fig. 2).

## Discussion

The most important findings of the present study are that, first, morphological cartilage imaging of the knee can be reliably performed using 3D TSE MRI, showing good image quality and high accuracy when compared to the standard of reference sequences. Second, although the newly available UTE sequence can distinguish between superficial and deep cartilage layers, it cannot be used as a single sequence to assess the articular cartilage at present.

The most common 3D sequences used in clinical practice to evaluate the articular cartilage of the knee are GRE-based methods, e.g. FLASH or DESS sequences. These sequences are available on most MR imaging systems and have been successfully used to evaluate cartilage. In addition, these sequences can be used to perform cartilage volume measurements in osteoarthritis research studies [4–8]. Although dark-fluid sequences have lower contrast between cartilage and fluid than bright-fluid sequences [4, 7], there was no significant difference in the performance of both sequences to evaluate cartilage in our study.

The development of balanced SSFP imaging, a variant of the GRE technique, was promising to improve 3D MRI of the musculoskeletal system. Several studies have shown excellent synovial fluid-cartilage contrast with these sequences. They are also useful in the imaging of other internal structures of the knee, such as the ligaments and menisci, a capability that makes it an attractive option for use in clinical practice [10, 20]. Although our study results confirm good contrast properties between cartilage and synovial fluid, the TruFISP sequence yielded more severe susceptibility artefacts and poorer overall image quality than did the conventional GRE methods.

Despite these developments, GRE imaging displays image contrast characteristics different from those of the TSE pulse sequences commonly used in assessment of joints. Recently, 3D TSE sequences (e.g. SPACE) have been used to assess the knee joint with high spin-echo contrast resolution and isotropic spatial resolution, improving conspicuity of cartilage lesions [5, 21]. Therefore, 3D TSE retains the advantages of 2D TSE while also addressing its limitations. The 3D TSE sequence typically uses variable flip angle modulation to constrain T2 decay over an extended echo train. This allows for intermediate-weighted images of the knee with bright fluid to be acquired with minimal blurring [11]. Our study results are in concordance with prior studies demonstrating superior performance of 3D SPACE in assessing cartilage lesions [14, 21]. A disadvantage of the SPACE sequence is its long acquisition time (10 min 4 s). This limitation, however, is counterbalanced by the fact that SPACE, due to its favourable tissue contrast, allows for comprehensive knee joint assessment. A single acquisition of 3D SPACE may therefore replace multiple 2D FSE acquisitions, which increases the time efficiency when used in a clinical knee MR protocol [21].

The osteochondral junction has been implicated in the pathogenesis of OA [22] and cartilage repair [23]. Thus, direct visualisation of these tissue components is clinically relevant. To date, MR imaging has, however, not been capable of assessing the deep radial and calcified layers of cartilage. These deep layers of cartilage have short T2 characteristics (<1 ms), and conventional pulse sequences are unable to acquire data in this range. UTE sequences are designed to target tissue components with very short T2 and allow signal to be detected in the deep layer of cartilage [24]. In a recent study, Bae et al. [18] compared UTE MRI and histology of experimental preparations and determined that the presence of the deepest layer of uncalcified cartilage and the calcified cartilage, but not the subschondral bone, results in this linear signal intensity in UTE MRI.

The term UTE imaging has generally been applied to techniques using shorter RF excitation pulses and faster readout methods than conventional methods to produce images with very short TEs, typically in the range of 8–250 μs [24]. A number of UTE techniques focussing on the method of image acquisition have been developed [25, 26]. These include both 2D and 3D sequences. They are typically combined with some form of long T2 component reduction in order to isolate the signal from the short T2 components and thus demonstrate change in disease [24].

Although a characteristic pattern of high linear signal near the osteochondral junction and low signal in the superficial cartilage layer could be observed in all subjects, sensitivity of our 3D UTE sequence in the detection of cartilage lesions was significantly lower compared to conventional sequences. Poor performance was probably related to blurring artefacts in the radial UTE acquisition together with field heterogeneity or potential gradient delays. Even at retrospective analysis, focal or diffuse abnormalities in the linear signal were found in only 10 of the 20 cartilage lesions. Most of these had high-grade cartilage lesions diagnosed on conventional MR sequences. In ten cartilage lesions, no abnormality in the UTE morphology of the osteochondral junction could be found. Of note, seven of these ten were apparent high-grade cartilage lesions on conventional MR sequences. This finding may suggest that these lesions were rather low-grade defects and overestimated on conventional MR images. Larger studies with arthroscopic correlation are needed to explore how the normal UTE signal pattern changes with disease and injury.

As with conventional MR techniques, UTE can also be used for quantitative mapping of tissues. A recent in vitro study [25] has evaluated UTE T2* and T1rho values of the patellar osteochondral junction in cadaveric samples and found that these measurements were useful for non-invasive assessment of the deep calcified layer of cartilage, including understanding the involvement of this tissue component in osteoarthritis.

There were several limitations to this study. First, and most important, the small number of patients limited the statistical analysis. However, in this era of limited resources and cost savings in health care, a larger study including more patients would not be possible in our busy clinical practice. Second, there was no arthroscopic correlation available. Great care was taken to obtain the best possible standard of reference using all available MR sequences and consensus readings. High accuracy of these sequences for detecting cartilage lesions is well documented in the literature [4–6]. Furthermore, arthroscopy also has limitations and should be considered an imperfect reference standard for grading of cartilage defects [27]. Third, all readers were employed in centres equipped with the sequences tested in our study; this may have introduced an interpretation bias, since the readers were very probably able to identify the sequence from the overall appearance of the blinded MR images. Fourth, in our study, we evaluated only subjective image quality, without quantitative analysis of the signal-to-noise (SNR) or contrast-to-noise (CNR) ratio. However, quantitative analysis of images acquired with parallel imaging requires that the “difference method” be used [28]. This would have been at the cost of doubling the total imaging time leading to motion artefacts again disturbing the methodological improvement.

## Conclusion

Our study, comparing various isotropic 3D sequences at 3 T, confirmed that 3D TSE MRI can be reliably performed for morphological cartilage imaging of the knee. In addition, further studies with arthroscopic correlation are needed to explore the role of clinical UTE sequences in the MR imaging assessment of cartilage lesions.
